# Mitochondrial D-Loop Region Methylation Is Not Altered in Children with Autism Spectrum Disorder

**DOI:** 10.3390/epigenomes10020025

**Published:** 2026-04-04

**Authors:** Andrea Stoccoro, Carmela Serpe, Antonia Parmeggiani, Vincenzo Davide Catania, Mario Lima, Alessandro Ghezzo, Cristina Panisi, Marida Angotti, Beatrice Pranzetti, Provvidenza Maria Abruzzo, Cinzia Zucchini, Lucia Migliore, Marina Marini, Fabio Coppedè

**Affiliations:** 1 Department of Translational Research and of New Surgical and Medical Technologies, University of Pisa, Via Roma, 55, 56126 Pisa, Italy; andrea.stoccoro@unipi.it (A.S.); lucia.migliore@unipi.it (L.M.); 2 Department of Medical and Surgical Sciences (DIMEC), University of Bologna, Via Massarenti, 9, 40138 Bologna, Italy; carmela.serpe@unibo.it (C.S.); provvidenza.abruzzo2@unibo.it (P.M.A.); cinzia.zucchini@unibo.it (C.Z.); marina.marini@unibo.it (M.M.); 3 IRCCS Istituto delle Scienze Neurologiche di Bologna, UOC Neuropsichiatria dell’Età Pediatrica, Via Altura, 3, 40139 Bologna, Italy; antonia.parmeggiani@unibo.it (A.P.); maridangotti@hotmail.it (M.A.); beatrice.pranzetti@studio.unibo.it (B.P.); 4 Pediatric Surgery Department, IRCCS Azienda Ospedaliero-Universitaria di Bologna, 40138 Bologna, Italy; vincenzodavide.catania@aosp.bo.it (V.D.C.); mario.lima@unibo.it (M.L.); 5 Fondazione Danelli ONLUS, Largo Stefano ed Angela Danelli 1, 26900 Lodi, Italy; a.ghezzo@fondazionedanelli.org; 6 Child Psychopathology Unit, Scientific Institute, IRCCS E. Medea, 23842 Bosisio Parini, Italy; panisi.cris@gmail.com

**Keywords:** autism spectrum disorder, epigenetics, mitochondrial DNA methylation, D-loop region, mitochondrial DNA copy number

## Abstract

**Background/Objectives**: Although the etiopathogenesis of autism spectrum disorder (ASD) remains incompletely elucidated, current evidence supports a multifactorial model involving genetic and environmental factors that interact to induce a heterogeneous range of symptoms. In recent years, epigenetic mechanisms, particularly DNA methylation, have been recognized as key contributors to ASD pathophysiology. Alterations in mitochondrial DNA (mtDNA) methylation are also emerging as relevant contributors in several human conditions. The mitochondrial D-loop, a non-coding control region essential for mtDNA replication and transcription, is considered a hotspot for epigenetic regulation and its methylation levels have been found altered in various diseases, such as cancer, metabolic disorders, and neurological illness. However, to date, no studies have investigated mtDNA methylation changes in ASD. **Methods**: We analyzed the average methylation levels of a fragment containing ten CpG sites within the D-loop region and the mtDNA copy number in peripheral blood samples from 49 children with ASD and 50 neurotypically developing (NT) controls using Methylation-Sensitive High-Resolution Melting and quantitative PCR. **Results**: No significant differences in D-loop methylation levels were observed between ASD and NT children. Similarly, the mtDNA copy number did not differ between the two groups. No significant correlations were found between D-loop methylation or mtDNA copy number and either ASD severity or age. **Conclusions**: This is the first study investigating mtDNA methylation in ASD. Our results indicate that methylation of the D-loop region and the mtDNA copy number are not altered in ASD children. Further studies including larger cohorts and extended mtDNA regions are warranted to confirm and expand these findings.

## 1. Introduction

Autism spectrum disorder (ASD) is a complex neurodevelopmental condition that usually manifests during early childhood and is characterized by deficits in social communication, repetitive behaviors, restricted interests, and altered sensory processing, including hypo- or hypersensitivity [1]. According to recent estimates, ASD affects approximately one in 127 individuals worldwide, with prevalence rates reaching up to one in 31 children in some U.S. cohorts [2,3]. However, the prevalence estimates may vary substantially depending on the diagnostic criteria, study design, and geographic region. Moreover, ASD is consistently reported to occur more frequently in males than in females, with a male-to-female ratio ranging from approximately 3:1 to 4:1 [4]. Although the first signs of ASD often emerge during the first two years of life, the diagnosis is usually made between three and five years of age, hindering the timely initiation of targeted interventions [5]. To date, the diagnosis of ASD is primarily based on clinical observation and standardized behavioral evaluation tools, and specific biological markers that could facilitate earlier and more accurate detection of the disorder are still lacking [6].

A better understanding of the biological basis of ASD is therefore essential for improving diagnostic precision that leads to preventive and therapeutic strategies. The etiopathogenesis of ASD is highly complex, and both genetic and environmental factors are known to contribute to its development [7]. The strong influence of genetic components is supported by the high heritability estimates reported in twin studies, ranging from 64% to 91% [8]. In addition, there are several genetic syndromes, such as tuberous sclerosis and Rett syndrome, that frequently present clinical ASD features [9]. The genetic architecture of ASD is now recognized to be extremely heterogeneous, involving a wide spectrum of genomic alterations, including single-nucleotide polymorphisms, copy number variants, and de novo mutations [10]. However, the identified or suspected genetic variants are estimated to account for only a minor part of ASD cases (about 10–20%), and in approximately 70% of cases, the disorder is likely shaped by complex interactions between multiple low-penetrance genetic variants and adverse environmental exposures [7,11]. However, the causal pathways linking such environmental influences to altered neurodevelopmental trajectories in ASD remain to be fully understood.

In this context, epigenetic regulation, particularly DNA methylation, has emerged as a crucial interface through which environmental factors can influence genome activity and contribute to ASD etiology [12]. Notably, several ASD risk genes encode proteins that participate in chromatin remodeling and epigenetic regulation, such as the *MECP2* gene, which encodes the methyl-CpG-binding protein 2, a key regulator of gene expression across the genome in Rett syndrome [13]. Moreover, in idiopathic ASD, aberrant DNA methylation patterns have been reported in animal ASD models and in ASD subjects [14]. Of note, altered DNA methylation levels have been detected by several authors in peripheral tissues of ASD subjects, such as saliva and peripheral blood, holding promise as a potential source of peripheral biomarkers for ASD [12]. Although growing evidence supports the potential diagnostic and prognostic relevance of DNA methylation patterns, their clinical applicability as reliable biomarkers for ASD diagnosis or symptom severity assessment remains to be established [15].

In recent years, evidence has suggested that not only nuclear DNA methylation but also mitochondrial DNA (mtDNA) methylation may contribute to the pathogenesis of various human diseases [16]. The mitochondrial genome is a circular DNA molecule that contains 37 genes essential for cellular energy metabolism, including 13 genes encoding proteins of oxidative phosphorylation, 22 genes for transfer RNAs, and 2 genes for ribosomal RNAs. The only non-coding region within the mtDNA, known as the displacement loop (D-loop), plays a regulatory role in mtDNA replication and transcription [17]. Several studies have indicated that methylation of the D-loop region can influence the mtDNA copy number and gene expression, and its dysregulation has been associated with multiple human disorders, including cancer, metabolic syndromes, and neurodegenerative diseases [18]. Of note, altered mtDNA methylation has been detected in peripheral blood cells of individuals with diseases of the central nervous system, such as Alzheimer’s disease [19,20], amyotrophic lateral sclerosis [21,22], bipolar disorder [23], and cerebral autosomal dominant arteriopathy with subcortical infarcts and leukoencephalopathy [24]. Such evidence suggests that peripheral blood could be a valuable source for mtDNA methylation biomarkers of brain disorders. Notably, mitochondrial dysfunction, including the abnormal activities of the electron transport chain and impaired energy metabolism, has long been implicated in ASD pathophysiology [25]. Increasing evidence supports a central role of mitochondrial abnormalities in ASD, to the extent that some authors have proposed the disorder as a mitochondria-related disease. These alterations may critically affect synaptic development, plasticity, and neuronal survival, processes that are essential during early brain development [25]. However, despite this evidence, no studies have specifically investigated mtDNA methylation in individuals with ASD.

The present study, therefore, aimed to assess D-loop methylation levels and mtDNA copy number in peripheral blood samples from male children with ASD and neurotypically developing (NT) controls to identify potential disease-related mitochondrial epigenetic alterations.

## 2. Results

### 2.1. D-Loop Methylation Levels in ASD and NT Children

**Figure 1:**
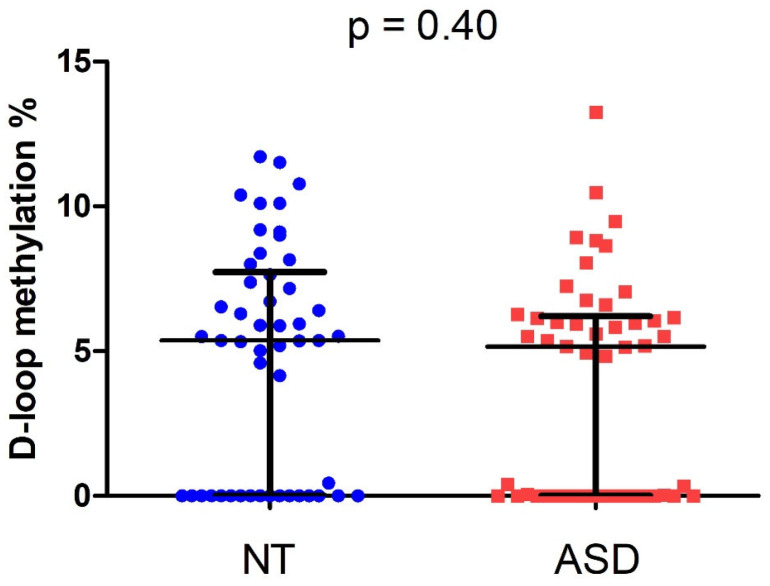
D-loop methylation levels in neurotypically developing (NT) and ASD children.

D-loop methylation levels were investigated by means of Methylation Sensitive High-Resolution Melting (MS-HRM) technique. MS-HRM analyses showed median D-loop methylation levels of 5.4% [IQR 0–7.7%] and 5.2% [IQR 0–6.2%] in NT and ASD children, respectively (Figure 1). The difference was not statistically significant (Mann–Whitney U = 1107.5, *p* = 0.40; r = 0.08).

**Figure 2:**
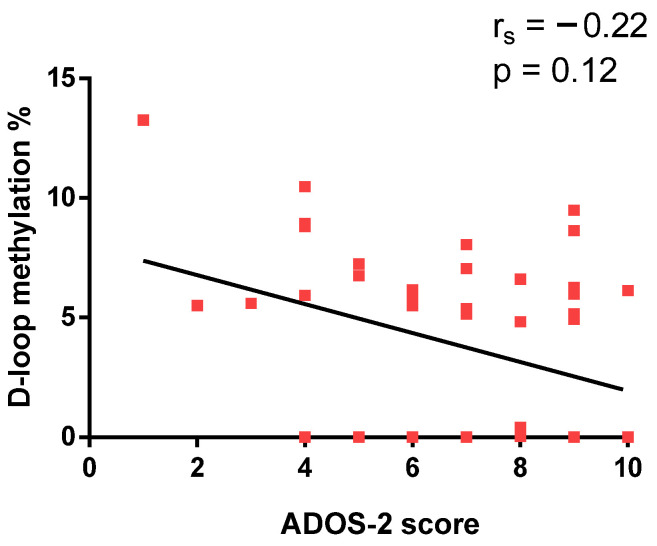
Correlation between D-loop methylation levels and ADOS-2 score.

To assess symptom severity, the Autism Diagnostic Observation Schedule, Second Edition (ADOS-2), was administered to ASD subjects. The ADOS-2 calibrated comparison score reflects ASD symptom severity on a scale from 1 (minimal) to 10 (highest). Spearman coefficient analysis revealed no significant correlation between ADOS-2 score and D-loop methylation levels (r_s_ = −0.22, *p* = 0.12, Figure 2).

### 2.2. mtDNA Copy Number in ASD and NT Children

**Figure 3:**
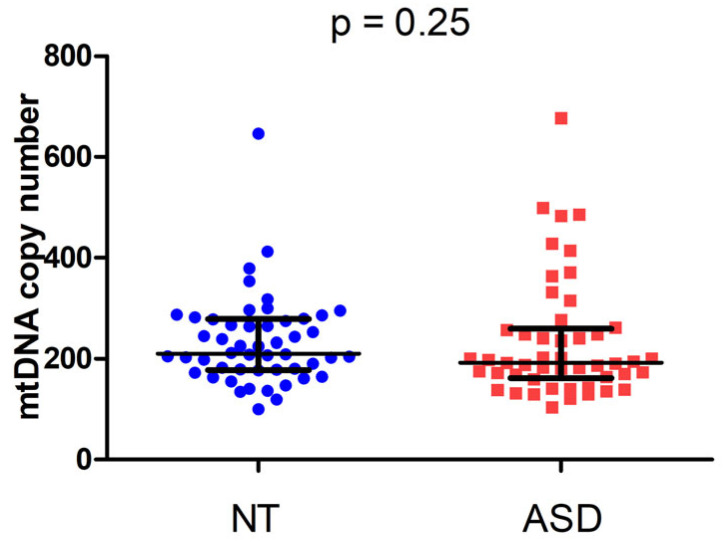
mtDNA copy number in neurotypically developing (NT) and ASD children.

We then searched for a potential correlation between mtDNA copy number and ASD status. The mtDNA copy number was quantified by real-time PCR using a mitochondrial target normalized to a single-copy nuclear gene. The mtDNA copy number did not significantly differ between NT (median 210.3, IQR 178.0–278.1) and ASD children (median 192.3, IQR 161.9–258.9) (Figure 3; Mann–Whitney U = 1063, *p* = 0.25; r = 0.11).

**Figure 4:**
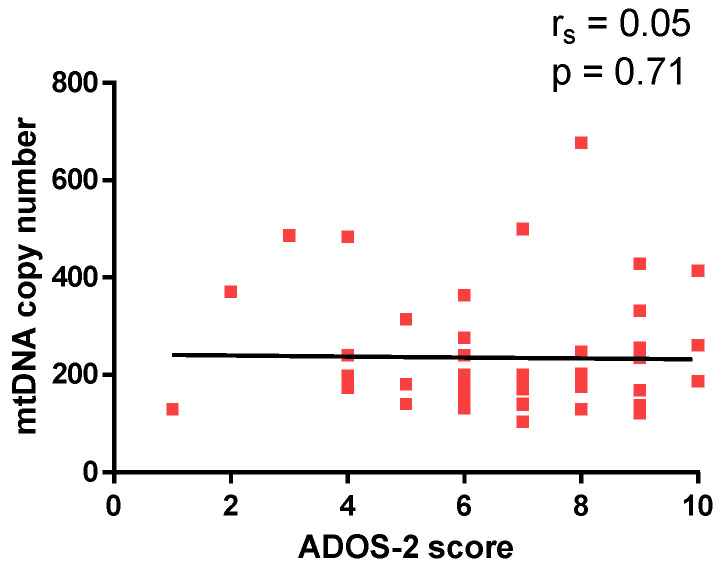
Correlation between mtDNA copy number and ADOS-2 score.

Moreover, Spearman coefficient analysis revealed no significant correlation between ADOS-2 score and mtDNA copy number (r_s_ = 0.05, *p* = 0.71, Figure 4).

### 2.3. Effects of Age on D-Loop Methylation and mtDNA Copy Number

**Figure 5:**
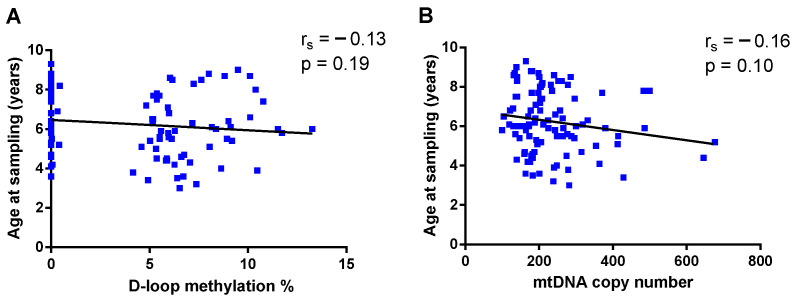
Effects of age at sampling on D-loop methylation (**A**) and mtDNA copy number (**B**).

To ensure that age at sampling did not affect DNA methylation and mtDNA copy number results, we examined possible associations among these variables. Neither D-loop methylation (r_s_ = −0.13, *p* = 0.19), nor mtDNA copy number (r_s_ = −0.16, *p* = 0.10) significantly correlated with age at sampling (Figure 5A and 5B, respectively).

### 2.4. Correlation Between D-Loop Methylation and mtDNA Copy Number

**Figure 6:**
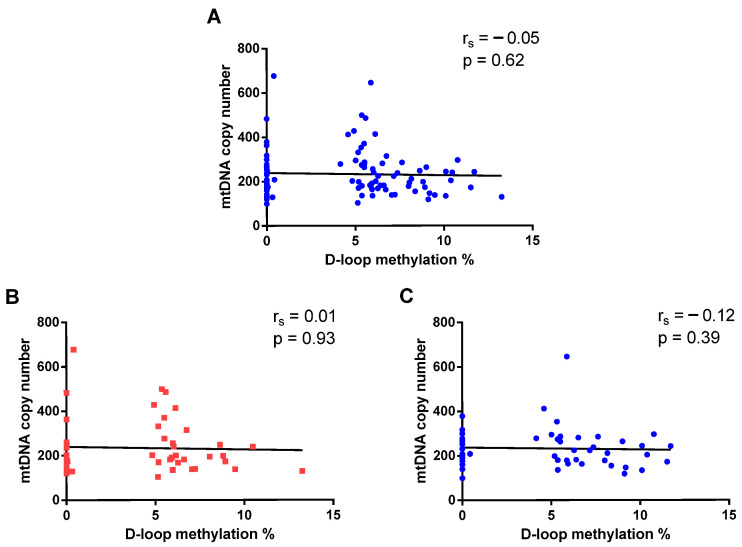
Correlation between mtDNA copy number and D-loop region methylation levels in total population (**A**), in ASD (**B**) and NT (**C**) groups.

Given the regulatory role of the mitochondrial D-loop region in mtDNA replication, we explored the potential relationship between D-loop methylation levels and mtDNA copy number in our cohort. As shown in Figure 6A, no significant correlation was observed between D-loop methylation and mtDNA copy number in total population (r_s_ = −0.05, *p* = 0.62). Similar results were obtained when ASD (Figure 6B) and NT (Figure 6C) groups were analyzed separately (ASD: r_s_ = 0.01, *p* = 0.93; NT: r_s_ = −0.12, *p* = 0.39).

## 3. Discussion

In the current study, we investigated mitochondrial D-loop methylation levels and mtDNA copy number in NT and ASD male children. Both D-loop methylation levels and mtDNA copy number did not significantly differ between the two groups. Moreover, neither D-loop methylation nor mtDNA copy number showed a significant correlation with disease severity, as measured by the ADOS-2 score. Age at sampling was not significantly correlated with either D-loop methylation or mtDNA copy number, and D-loop methylation levels did not correlate with mtDNA copy number.

The etiology of ASD is complex, and it is emerging that, in addition to genetic and environmental factors, altered epigenetic mechanisms play an important role in ASD. Indeed, epigenetic modifications are critical in orchestrating neurodevelopment. Disruptions in the mechanisms regulating these modifications during neurogenesis can lead to neurological disorders such as ASD. Altered levels of DNA methylation, often associated with the severity of the condition, have been found in individuals with ASD [26,27]. Moreover, altered methylation levels of genes involved in mitochondrial metabolism have been reported, suggesting an association between DNA methylation and mitochondrial dysfunction in the etiology of ASD [28,29]. However, studies investigating mtDNA methylation levels in ASD have not yet been conducted. Therefore, the present study is the first to examine mitochondrial D-loop methylation alterations in individuals with ASD. We focused on the mitochondrial D-loop region, as it is the only non-coding region of the mitochondrial genome that plays a pivotal role in mtDNA regulation, as it governs both mtDNA transcription and replication. Of note, DNA methyltransferases can interact with the mitochondrial D-loop region, where most of the methylated cytosines within the mitochondrial genome are localized. This suggests that methylation at this locus might play a regulatory role in mitochondrial gene expression and replication [30,31]. Although global mtDNA methylation levels are generally low, typically ranging between 0.5% and 1%, the D-loop region displays markedly higher methylation levels, with reported peaks of up to 10–15% [32,33]. Notably, methylation within the D-loop has been shown to influence the binding affinity of mitochondrial transcription factor A (TFAM), a central component in the control of mtDNA transcription and replication [34]. Moreover, several studies have reported that D-loop methylation levels correlate with mtDNA copy number [35,36,37,38] as well as with the expression of mitochondrial genes [37,38]. Previous research has found associations between D-loop methylation levels and several human diseases, including various types of cancer [39,40], metabolic disorders [41], neurodegenerative diseases [19,21,42] and ADHD [43]. Interestingly, D-loop methylation levels have been shown to be sensitive not only to the presence of disease but also to its progression. For example, in Alzheimer’s disease (AD), altered D-loop methylation has been observed in brain specimens from an AD mouse model and in the peripheral blood of living patients and appears to be sensitive to the stage of the pathology [19,44]. Indeed, multiple lines of evidence suggest that D-loop methylation levels are modulated during the progression of the pathology, showing increased levels in the prodromal stages of the disease and decreased levels in advanced stages [44,45]. Furthermore, D-loop methylation differed between patients in the prodromal stage of the pathology who convert to AD and those who do not [20]. These findings suggest that D-loop methylation levels in peripheral blood could reflect the status of brain disorders, potentially serving as peripheral biomarkers to identify patients along the spectrum of the pathology. However, in the current study, methylation levels of the D-loop segment analyzed were not altered in ASD subjects. Moreover, D-loop methylation was not correlated with the severity of the disease evaluated with the ADOS-2 score. So, the results of this study suggest that this specific D-loop region is not sensitive to ASD status. It should be noted that we enrolled only male subjects and that in females, the results could differ. Indeed, differences in D-loop methylation levels between sexes have been reported in the brain and peripheral blood of control subjects [33,46], and sex-specific nuclear DNA methylation signatures for ASD have also been identified [29,47].

We also investigated mtDNA copy number in ASD and NT children. The mtDNA copy number reflects the abundance of mitochondria within cells and can vary according to the energy requirements of the tissues [48]. Several studies have used mtDNA copy number as an indirect indicator of mitochondrial function, and its assessment has been proposed as a biomarker for various human diseases, including cancer, neurodegenerative disorders, and psychiatric conditions [49,50,51]. Given the pivotal role of mitochondria in energy production, calcium homeostasis, and overall cellular metabolism, mitochondrial impairment during neurogenesis has been suggested as a major contributor to ASD etiology [25]. Consistent with this, several biomarkers of mitochondrial dysfunction, such as mitochondrial metabolites, mtDNA variations, and mitochondrial activity, have been found to be altered in individuals with ASD [52]. However, evidence regarding mtDNA copy number alterations in ASD remains conflicting, as both increased, decreased, and unaltered levels of mtDNA copy number have been reported in the peripheral blood of ASD subjects compared with controls [52,53]. In the present study, we did not observe significant differences in mtDNA copy number levels between ASD and NT children. Moreover, mtDNA copy number did not correlate with the ADOS-2 score, revealing no association with disease severity. In light of the conflicting reports in the literature, our results support the view that mtDNA copy number changes, if present, may occur only in specific ASD subgroups or under particular environmental or developmental conditions. Further studies are needed to better elucidate the potential usefulness of mtDNA copy number evaluation in ASD subjects.

Given the well-established role of the mitochondrial D-loop region in regulating mtDNA transcription and replication [16], we explored the relationship between D-loop methylation and mtDNA copy number in our cohort. No significant correlation was observed between methylation levels of the analyzed D-loop region and mtDNA copy number, either in the overall population or when ASD and NT groups were examined separately. Previous studies performed in cell cultures and human samples have reported associations between D-loop methylation and mtDNA copy number, supporting a potential mechanistic link [39,46,54]. Conversely, other investigations did not detect a significant association between these two parameters [41,55], suggesting that this relationship may emerge only under specific biological conditions. Indeed, mitochondrial biogenesis and mtDNA replication are regulated by multiple nuclear and mitochondrial factors, including TFAM- and PGC-1α-mediated pathways [56], indicating that D-loop methylation likely represents only one layer within a complex regulatory network. Overall, our findings support the hypothesis that the relationship between mitochondrial epigenetic modifications and mtDNA copy number is context- and disease-dependent.

We acknowledge that this study has certain limitations. First, D-loop methylation was assessed using the MS-HRM technique, which provides an overall methylation estimate for the 10 CpG sites included in the amplified fragment rather than site-specific methylation values. Therefore, it remains possible that individual CpG sites within this region exhibit subtle methylation differences between ASD and NT children that cannot be resolved with this approach. Nevertheless, we employed a well-validated protocol that our group has previously used to successfully detect disease-related methylation differences in patients with Alzheimer’s disease, mild cognitive impairment, and amyotrophic lateral sclerosis [19,45,46,57]. Importantly, we also demonstrated that D-loop methylation levels quantified using this MS-HRM protocol are highly consistent with those obtained through pyrosequencing, the gold-standard method for locus-specific methylation assessment [58]. Moreover, our analysis was limited to a single region of the mitochondrial genome. Additional studies examining the entire mtDNA methylome, ideally with sequencing-based approaches capable of resolving CpG-specific variability, will be necessary to definitively determine whether mitochondrial methylation differences exist between ASD and control groups. Moreover, mtDNA copy number was estimated using a SYBR Green–based qPCR approach, which is widely used for relative quantification; nonetheless, future studies may consider probe-based assays or droplet digital PCR to further improve analytical specificity and precision. Another limitation of the present study is the absence of mitochondrial gene expression data. Given the well-established role of D-loop methylation in regulating mtDNA transcription and replication, such information would have contributed additional functional insight. Although we did not observe a correlation between D-loop methylation and mtDNA copy number in our cohort, it remains of interest to determine whether mitochondrial genes might be differentially expressed between ASD and NT children, even in the absence of detectable methylation differences. Future studies integrating CpG-specific mtDNA methylation profiles, mtDNA copy number measurements, and mtDNA gene expression analyses will be essential to provide a more comprehensive understanding of mitochondrial regulatory mechanisms in ASD. It should also be mentioned that only male subjects were included in the present study, which, although it helped in reducing the variability due to sex-related epigenetic differences, did not allow us to obtain information regarding female ASD subjects. We also acknowledge that the sample size was relatively small, and the present findings should be confirmed in larger and independent cohorts. Finally, methylation analyses were performed exclusively in peripheral blood. Although blood represents a readily accessible surrogate tissue in pediatric studies, mitochondrial epigenetic regulation may be tissue-specific, and therefore, findings obtained in peripheral samples may not fully reflect mitochondrial regulatory mechanisms occurring in the brain.

In summary, this pilot investigation examined possible changes in mitochondrial D-loop methylation and mtDNA copy number in individuals with ASD. The data obtained do not indicate significant differences in either parameter when compared with sex- and age-matched controls, nor were associations observed with ASD severity. Importantly, to our knowledge, this is the first study to investigate mtDNA methylation in ASD, providing preliminary evidence that no detectable alterations are present in the analyzed D-loop fragment in peripheral blood within this cohort. Establishing what is not affected is essential for delineating the specific mitochondrial pathways that may or may not contribute to ASD pathophysiology. Although mitochondrial abnormalities are frequently reported in ASD, the present findings suggest that these dysfunctions are not mirrored by alterations in methylation of the analyzed D-loop region in peripheral blood. Additional studies, including larger populations and broader coverage of the mitochondrial genome, will be required to corroborate and extend these results.

## 4. Materials and Methods

### 4.1. Study Population

Participants, all male children aged 4 to 8 years, were recruited within the framework of a research project funded by FIA-Fondazione Italiana per l’Autismo ETS (Italian Foundation for Autism ETS) and by the Associazione per la ricerca sull’autismo Cimadori OdV ETS (Association Cimadori for autism research OdV ETS). Two demographically homogeneous groups were selected: the first group included 49 children diagnosed with idiopathic autism spectrum disorder (ASD, mean age = 6.3 ± 1.7 years), and the second group comprised 50 neurotypically developing children (NT, mean age = 6.2 ± 1.5 years). The NT children were enrolled from the Pediatric Surgery Department among patients undergoing minimally invasive procedures, the ASD subjects were recruited at the IRCCS Istituto delle Scienze Neurologiche of Bologna, Italy.

Children included in the study had a confirmed clinical diagnosis of Autism Spectrum Disorder (ASD) established according to the criteria of the Diagnostic and Statistical Manual of Mental Disorders, Fifth Edition (DSM-5) [59]. To confirm the diagnosis and assess symptom severity, the Autism Diagnostic Observation Schedule, Second Edition (ADOS-2) [60], was administered. The ADOS-2 provides standardized measures of social communication and restricted/repetitive behaviors, yielding a calibrated comparison score ranging from 1 (minimal symptom severity) to 10 (highest symptom severity). Moreover, ASD children admitted in the study had negative results on CGH array testing. Exclusion criteria for both groups included neurological disorders, epilepsy, ongoing or recent infectious diseases (within the last 4 months), recent surgery or trauma (within the last 4 months), and the use of supplements or vitamins in the month prior to sample collection. Peripheral blood samples were collected for each participant, and an aliquot was immediately stored at −80 °C until analysis. The study was conducted in accordance with the principles of the Declaration of Helsinki and its subsequent amendments or equivalent ethical standards. The study was approved by the Ethics Committee of Area Vasta Centro (CE AVEC number 849-2021-OSS-AUSLBO-SIRER ID 2897) on 21 October 2021, and written informed consent was obtained from the parents or legal guardians of all participants before enrolment.

### 4.2. D-Loop Methylation Analysis

D-loop methylation was evaluated as previously reported [19,57]. Genomic DNA was isolated from peripheral whole blood collected in EDTA tubes using the QIAmp DNA Blood Mini Kit (Qiagen, Milan, Italy). DNA concentration and purity were assessed with a NanoDrop ND-2000c spectrophotometer (Thermo Scientific, Wilmington, DE, USA). For each subject, 200 ng of DNA were bisulfite converted using the EpiTect Bisulfite Kit (Qiagen, Milan, Italy), following the manufacturer’s instructions.

D-loop methylation levels were quantified using a Methylation-Sensitive High-Resolution Melting (MS-HRM) approach, which involves PCR amplification of the bisulfite-converted region followed by high-resolution melting analysis. PCR reactions were carried out with the following cycling conditions: an initial denaturation at 95 °C for 5 min, followed by 50 cycles at 95 °C for 30 s, 56 °C for 45 s, and 72 °C for 45 s. Primers were designed using the MethPrimer tool based on the GenBank reference sequence J01415.2. The sequences were: forward 5′-GGAGTTTTTTATGTATTTGGTATTTT-3′ and reverse 5′-ACAAACATTCAATTATTATTATTATATCCT-3′. This primer pair amplifies a 222-bp fragment of the mitochondrial D-loop (nucleotides 35–256 of J01415.2), encompassing 10 CpG sites. The analyzed fragment within the D-loop mitochondrial region was selected because it encompasses a CpG-rich segment that has been previously investigated in the context of human diseases and environmental exposures [19,44,61,62], supporting its biological relevance for methylation studies. Melting curve analysis was performed with the following protocol: denaturation at 95 °C for 10 s, annealing at 50 °C for 60 s, and gradual temperature increase from 65 °C to 95 °C at a rate of 0.2 °C every 15 s. All samples were analyzed in duplicate and each experiment was repeated in at least two independent runs.

For every MS-HRM assay, a standard curve was generated using mixtures of commercially available fully methylated and unmethylated DNA (Qiagen) to obtain reference samples ranging from 0% to 100% methylation. The standard curves generated from methylated and unmethylated DNA mixtures confirmed consistent amplification performance and excluded preferential amplification bias between methylated and unmethylated templates (Supplementary Figure S1). Methylation percentages for each sample were then calculated by interpolation of melting profiles using a custom MATLAB function (MATLAB R2020a, The MathWorks, Natick, MA, USA). Details on protocol set-up showing lack of preferential amplification of methylated vs. unmethylated templates, standard curves, and lack of non-specific amplification are provided as Supplementary Materials.

In our previous work, we demonstrated that this MS-HRM protocol performs equally well on both linearized and non-linearized mtDNA, as D-loop methylation levels obtained after enzymatic linearization overlapped with those measured in circular mtDNA [57]. Primer specificity was verified by melting curve analysis, showing a single amplification peak, as well as by agarose gel electrophoresis, demonstrating a single amplicon of the expected size (~222 bp) (Supplementary Figure S1). In our previous study [58], Sanger sequencing confirmed the correct amplification of the intended D-loop fragment and excluded the presence of sequence variants capable of altering melting behavior. Moreover, the MS-HRM procedure adopted here has been shown to generate D-loop methylation values highly consistent with those obtained using pyrosequencing, a gold-standard method for locus-specific methylation assessment [58].

### 4.3. mtDNA Copy Number Analysis

The mtDNA copy number was determined by quantitative PCR, as previously described [58]. For each sample, 10 ng of genomic DNA were used to simultaneously amplify a mitochondrial target (chrM:3313–3322) and a single-copy nuclear reference gene (hemoglobin subunit β). The ratio between the amplification signals of the two loci was used to estimate the relative abundance of mtDNA. qPCR reactions were run on a CFX96 Real-Time PCR Detection System (Bio-Rad, Hercules, CA, USA) with the following cycling protocol: an initial activation at 95 °C for 15 min, followed by 40 cycles of 95 °C for 30 s, 55 °C for 45 s, and 72 °C for 45 s. Reactions were prepared in 10 µL volume containing 5 µL of SYBR Green master mix (Qiagen, Milan, Italy), 10 pmol of each primer, and 1 µL of DNA template. All samples were analyzed in triplicate.

Ct values were obtained using the CFX Manager software v3.1 (Bio-Rad, Hercules, CA, USA), and relative mtDNA copy number was calculated using the ΔCt method: ΔCt = Ct_nDNA_ − Ct_mtDNA_; relative mtDNA copy number = 2 × 2^ΔCt^.

### 4.4. Statistical Analyses

The distribution of D-loop methylation levels and mitochondrial DNA (mtDNA) copy number was examined using the Shapiro–Wilk test to assess data normality. As both variables showed significant deviations from a normal distribution, non-parametric statistical procedures were adopted. Comparisons between two independent groups were carried out using the Mann–Whitney U test, with data shown as individual points along with the median and interquartile range (25th–75th percentile). Effect sizes (r) for Mann–Whitney comparisons were calculated as r = Z/√N, where Z is the standardized test statistic, and N is the total sample size. Correlations between continuous variables were evaluated using Spearman’s rank correlation coefficient. All statistical analyses were conducted using the STATGRAPHICS Plus 5.1 software package for Windows (Statgraphics Technologies, Inc., The Plains, VA, USA), whereas figures and graphical outputs were generated with GraphPad Prism, version 6.01 (GraphPad Software, San Diego, CA, USA).

## 5. Conclusions

This pilot study investigated mitochondrial D-loop methylation and mtDNA copy number in children with ASD compared with age- and sex-matched controls. No significant differences were observed in either parameter, and no association with ASD severity was detected. To our knowledge, this is the first study assessing mitochondrial DNA methylation in ASD, providing initial evidence that mitochondrial epigenetic regulation within the analyzed D-loop fragment in peripheral blood does not show detectable alterations in this cohort of ASD subjects.

However, several limitations should be acknowledged. Only a single D-loop fragment containing 10 CpG sites was examined using MS-HRM, which provides average methylation levels rather than site-specific resolution. Other regions of the mitochondrial genome were not analyzed, and methylation patterns were assessed exclusively in peripheral blood, which may not fully reflect brain-specific mitochondrial epigenetic regulation. In addition, only male subjects were included, limiting generalizability to females.

Larger studies incorporating broader mitochondrial genome coverage, site-specific methylation analyses, inclusion of female participants, and, where feasible, disease-relevant tissues will be necessary to further clarify the role of mitochondrial epigenetic mechanisms in ASD.

## Data Availability

The datasets generated and/or analyzed during the current study are available from the corresponding author on reasonable request.

## Supplementary Materials

The following supporting information can be downloaded at https://www.mdpi.com/article/10.3390/epigenomes10020025/s1: Figure S1: (A) Amplification curves of fully methylated (red) and fully unmethylated (green) standard DNA samples. (B) Amplification curves of fully methylated (red), fully unmethylated (green) standard DNA samples, samples with 0% (aqua green), ~5% (light green), and ~10% (dark blue) of methylation levels. (C) Melting curves generated by samples with known (0%, 12.5%, 25%, 50%, 75%, and 100%) methylation levels. (D) Melting curves generated by samples with known methylation levels, and of samples with 0% (aqua green), ~5% (light green), and ~10% (dark blue) methylation levels. (E) Electrophoresis gel visualization of the standard DNA completely unmethylated (U), the standard DNA completely methylated (M), a sample of DNA with 0% of methylation (0% S), a sample of DNA with ~5% of methylation (~5% S) and a sample of DNA with ~10% of methylation (~10% S); Table S1: Dataset used for the analyses performed in this study.

## Abbreviations

The following abbreviations are used in this manuscript:

ADOS-2	Autism Diagnostic Observation Schedule, Second Edition
ASD	Autism Spectrum Disorder
D-loop	Displacement-Loop
DSM-5	Diagnostic and Statistical Manual of Mental Disorders, Fifth Edition
mtDNA	Mitochondrial DNA
NT	Neurotypically Developing
