# Supplementary material for: Mitochondrial D-Loop Region Methylation Is Not Altered in Children with Autism Spectrum Disorder
Source: Epigenomes. 2026 Apr 4;10(2):25. doi: 10.3390/epigenomes10020025 (PMC13108014; doi:10.3390/epigenomes10020025)
Supplement: Supplementary file 1 [file epigenomes-10-00025-s001.zip › epigenomes-4137601-supplementary.pdf]

Mitochondrial D-Loop Region Methylation Is Not Altered in Children with Autism Spectrum Disorder

Supplementary Material

The specificity and amplification efficiency of the primers were first assessed using fully methylated and fully unmethylated control DNA. Both standards showed overlapping amplification curves and identical Ct values, indicating the absence of preferential amplification of either methylated or unmethylated templates (Supplementary Fig. S1A). Similarly, samples displaying different methylation levels (0%, ~5% and ~10%) also showed comparable amplification curves and identical Ct values, excluding differential amplification efficiency among samples with different methylation profiles (Supplementary Fig. S1B). The ability of the MS-HRM assay to discriminate different methylation levels was confirmed using standard curves generated from mixtures of methylated and unmethylated DNA (0%, 12.5%, 25%, 50%, 75% and 100%), which produced clearly distinguishable melting profiles (Supplementary Fig. S1C).

Consistently, samples with 0%, ~5% and ~10% methylation displayed melting curves coherent with their estimated methylation levels (Supplementary Fig. S1D).

Finally, agarose gel electrophoresis of PCR products showed a single band of the expected size (222 bp) in both standards and representative samples, confirming primer specificity and the absence of nonspecific amplification products (Supplementary Fig. S1E).

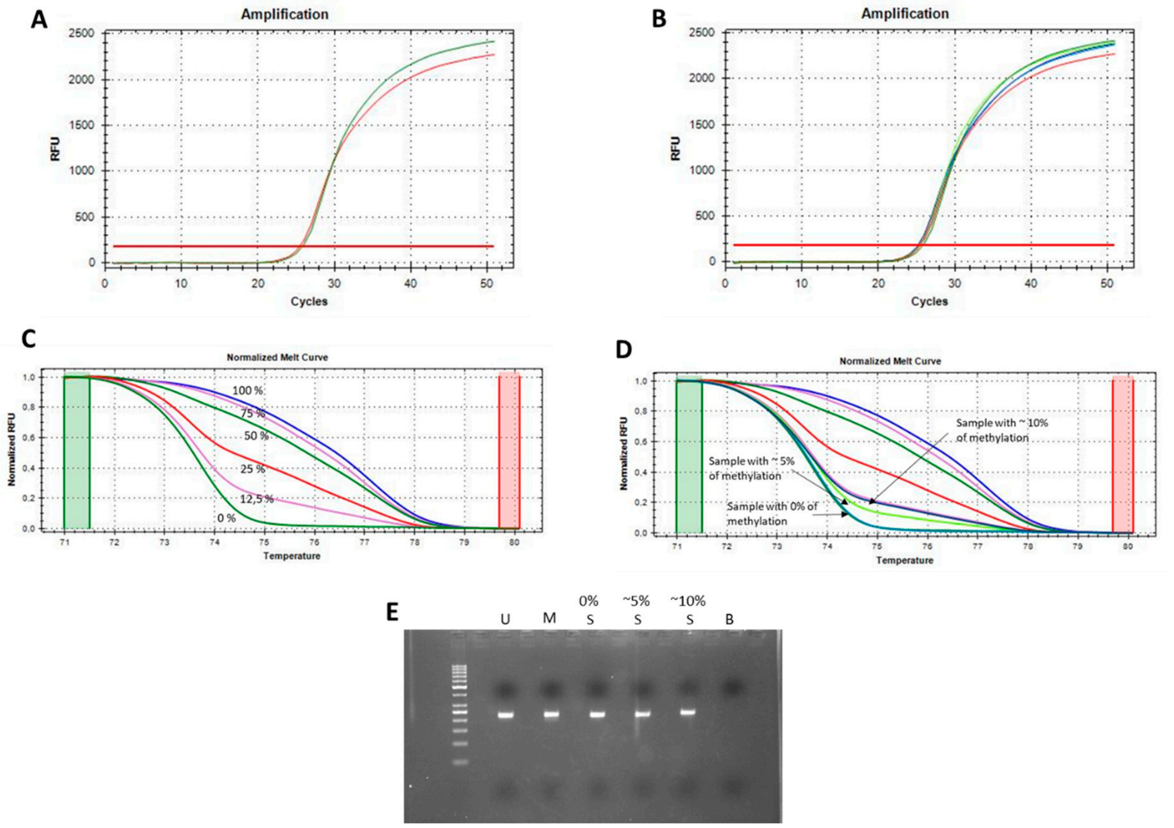

Supplementary Figure S1. A) Amplification curves of fully methylated (red) and fully unmethylated (green) standard DNA samples. B) Amplification curves of fully methylated (red), fully unmethylated (green) standard DNA samples, samples with 0% (aqua green), ~5% (light green), and ~10% (dark blue) of methylation levels. C) Melting curves generated by samples with known (0%, 12,5%, 25%, 50%, 75%, and 100%) methylation levels. D) Melting curves generated by samples with known methylation levels, and of samples with 0% (aqua green), ~5% (light green), and ~10% (dark blue) methylation levels. E) Electrophoresis gel visualization of the standard DNA completely unmethylated (U), the standard DNA completely methylated (M), a sample of DNA with 0% of methylation (0% S), a sample of DNA with ~5% of methylation (~5% S) and a sample of DNA with ~10% of methylation (~10% S).

Supplementary Table S1. Dataset used for the analyses performed in this study

ID code	Age (years)	ADOS-2 score[1]	D-loop methylation levels (%)	mtDNA Copy Number
**ASD 1**	7,8	9	0,00	235,48
**ASD 2**	5,9	3	5,59	486,34
**ASD 3**	5,2	8	0,41	676,71
**ASD 4**	5,6	6	5,51	276,64
**ASD 5**	7,8	7	5,37	499,75
**ASD 6**	5,5	10	6,13	414,52
**ASD 7**	7,7	2	5,50	370,80
**ASD 8**	4,1	6	0,00	363,66
**ASD 9**	5,5	8	0,03	248,20
**ASD 10**	3,4	9	4,93	428,45
**ASD 11**	7,8	4	0,00	483,47
**ASD 12**	6,8	9	5,99	256,60
**ASD 13**	4,4	6	5,83	182,69
**ASD 14**	3,9	4	10,48	240,03
**ASD 15**	7,2	8	4,83	202,59
**ASD 16**	4,3	7	7,05	139,13
**ASD 17**	8,6	6	6,04	240,51
**ASD 18**	6,5	7	5,14	103,58
**ASD 19**	6,5	7	5,18	170,24
**ASD 20**	4,2	8	0,06	175,48
**ASD 21**	6,8	8	0,00	203,00
**ASD 22**	6,9	8	0,33	129,65
**ASD 23**	5,6	6	5,97	135,99
**ASD 24**	3,6	7	0,00	200,83
**ASD 25**	8,5	6	6,15	200,69
**ASD 26**	9,3	6	0,00	164,25
**ASD 27**	4,2	9	6,27	169,13
**ASD 28**	4,6	8	6,61	182,44
**ASD 29**	6,0	1	13,26	129,81
**ASD 30**	8,8	5	0,00	180,89
**ASD 31**	7,1	4	5,93	190,80
**ASD 32**	5,3	8	0,00	192,30
**ASD 33**	4,0	9	8,64	248,42
**ASD 34**	4,7	5	6,76	314,90
**ASD 35**	8,3	5	7,24	140,43
**ASD 36**	6,3	9	5,16	331,66
**ASD 37**	8,3	10	0,00	261,21
**ASD 38**	6,8	9	0,00	121,13
**ASD 39**	8,6	6	0,00	131,55
**ASD 40**	8,5	7	0,00	140,75
**ASD 41**	8,7	4	8,82	198,42
**ASD 42**	5,5	4	8,93	173,63

**ASD 43**	9,0	9	9,49	138,36
**ASD 44**	5,1	7	8,05	195,32
**ASD 45**	4,6	6	0,00	159,64
**ASD 46**	6,1	8	0,00	172,47
**ASD 47**	4,7	10	0,00	187,04
**ASD 48**	6,0	6	0,00	142,96
**ASD 49**	6,2	6	0,00	188,49
**NT 1**	4,4	-	5,89	646,05
**NT 2**	5,0	-	5,33	353,80
**NT 3**	5,9	-	6,29	225,12
**NT 4**	3,8	-	4,16	278,89
**NT 5**	5,9	-	0,00	379,32
**NT 6**	5,4	-	5,02	295,15
**NT 7**	5,5	-	5,51	263,86
**NT 8**	8,3	-	0,00	252,19
**NT 9**	8,5	-	7,64	285,70
**NT 10**	7,7	-	5,37	180,01
**NT 11**	8,1	-	0,00	231,63
**NT 12**	5,1	-	4,60	412,61
**NT 13**	4,5	-	5,36	274,66
**NT 14**	5,2	-	0,00	205,10
**NT 15**	7,5	-	5,37	136,03
**NT 16**	5,8	-	5,95	164,08
**NT 17**	3,5	-	6,40	182,43
**NT 18**	5,5	-	5,52	287,12
**NT 19**	4,5	-	5,88	179,06
**NT 20**	4,7	-	0,00	161,00
**NT 21**	8,1	-	0,00	277,87
**NT 22**	3,6	-	6,72	163,16
**NT 23**	6,1	-	0,00	317,83
**NT 24**	6,8	-	0,00	177,02
**NT 25**	3,0	-	6,53	281,61
**NT 26**	7,7	-	5,20	198,22
**NT 27**	6,1	-	8,16	211,45
**NT 28**	8,8	-	8,01	178,26
**NT 29**	8,0	-	0,00	202,00
**NT 30**	6,0	-	0,00	299,61
**NT 31**	6,3	-	7,17	224,41
**NT 32**	3,2	-	7,38	238,28
**NT 33**	8,2	-	0,44	209,17
**NT 34**	6,4	-	0,00	190,08
**NT 35**	5,9	-	0,00	266,20
**NT 36**	6,0	-	8,38	155,21
**NT 37**	7,4	-	10,78	296,05
**NT 38**	6,0	-	11,52	172,14

**NT 39**	7,5	**-**	0,00	140,45
**NT 40**	5,4	**-**	9,19	146,72
**NT 41**	8,0	**-**	10,40	204,58
**NT 42**	8,7	**-**	10,10	134,17
**NT 43**	6,6	**-**	10,11	244,22
**NT 44**	8,1	**-**	0,00	206,86
**NT 45**	6,4	**-**	9,01	264,17
**NT 46**	7,4	**-**	0,00	202,78
**NT 47**	7,4	**-**	0,00	208,35
**NT 48**	6,1	**-**	9,12	119,04
**NT 49**	5,8	**-**	0,00	99,92
**NT 50**	5,8	**-**	11,72	242,91

[1]ADOS2: Autism Diagnostic Observation Schedule - Version 2
